# FairCs—Blockchain-Based Fair Crowdsensing Scheme using Trusted Execution Environment

**DOI:** 10.3390/s20113172

**Published:** 2020-06-03

**Authors:** Yihuai Liang, Yan Li, Byeong-Seok Shin

**Affiliations:** Department of Electrical and Computer Engineering, Inha University, 100 Inha-ro, Michuhol-gu, Incheon 22212, Korea; liangyhgood@inha.edu (Y.L.); leeyeon@inha.ac.kr (Y.L.)

**Keywords:** crowdsensing, blockchain, trusted execution environment, fairness, security

## Abstract

Crowdsensing applications provide platforms for sharing sensing data collected by mobile devices. A blockchain system has the potential to replace a traditional centralized trusted third party for crowdsensing services to perform operations that involve evaluating the quality of sensing data, finishing payment, and storing sensing data and so forth. The requirements which are codified as smart contracts are executed to evaluate the quality of sensing data in a blockchain. However, regardless of the fact that the quality of sensing data may actually be sufficient, one key challenge is that malicious requesters can deliberately publish abnormal requirements that cause failure to occur in the quality evaluation process. If requesters control a miner node or full node, they can access the data without making payment; this is because of the transparency of data stored in the blockchain. This issue promotes unfair dealing and severely lowers the motivation of workers to participate in crowdsensing tasks. We (i) propose a novel crowdsensing scheme to address this issue using Trusted Execution Environments; (ii) offer a solution for the confidentiality and integrity of sensing data, which is only accessible by the worker and corresponding requester; (iii) and finally, report on the implementation of a prototype and evaluate its performance. Our results demonstrate that the proposed solution can guarantee fairness without a significant increase in overhead.

## 1. Introduction

### 1.1. Blockchain-Based Crowdsensing Application and Challenges

Crowdsensing [1,2,3] is a technique where a group of individuals with mobile devices, capable of sensing and computing collectively share data and extract information, to measure, map, analyze, estimate, or infer any processes of common interest. Traditional centralized crowdsensing platform services belong to companies or other organizations, which may not be 100% trustworthy [4]. For instance, they may collude with requesters to trick workers, so that workers gain less rewards. Furthermore, their service may break off because of single point failure, causing poor service availability and loss of user data. In addition, sensing data usually contain privacy information such as user location. It remains a concern that the privacy of workers could be exploited and leaked by malicious providers of a crowdsensing service. For example, providers could analyze all location data of a specified user to obtain the user’s mobile trajectory.

A blockchain could replace the role of traditional centralized crowdsensing service providers. First, blockchain can provide anonymity (or pseudonymity precisely), referring to the non-identifiability of the sender and the receiver in a single transaction. Although, currently, the pseudo identities of users in a blockchain are not completely unlinkable with their real identities [5,6], many solutions have been proposed to enhance the pseudonymity of blockchain, such as Zerocoin [7], Zerocash [8], zk-SNARK [9], CoinJoin [10]. Second, a blockchain-based crowdsensing platform can evaluate sensing data based on the requesters’ requirements through a smart contract [11] in the blockchain. Majority nodes need to successfully validate and approve the fact that the sensing data meet the requirements before adding the transaction to the blockchain. Third, the rewards for workers can be paid automatically and mandatorily by smart contracts if the data meet requirements. Therefore, workers do not need to be concerned about receiving unfair rewards if the sensing data meet the requirements, likewise requesters do not need to be concerned about obtaining low quality sensing data post payment. In short, applying blockchain techniques to crowdsensing applications is an attractive and promising prospect [12,13] to ensure that the service of crowdsensing transactions is highly available, privacy-preserved, and fair.

However, many challenges still remain in applying blockchain to crowdsensing applications. First, blockchain can guarantee fairness for a crowdsensing transaction by replicating the validation of smart contract transactions in each peer node. However, the requirements of sensing data are created and published by requesters, who can maliciously create abnormal smart contracts of requirements to maximize their profits. Second, sensing data may contain the privacy information of workers; however, everyone has access to the information because of the openness and transparency of contract execution in the blockchain. This causes great concern in terms of privacy leakage and results in a reduction in the interest of workers to participate in crowdsensing tasks. Third, if the size of the crowdsensing data is large, it is difficult to transmit and store data over the whole blockchain of peers, because this results in a significant amount of network and storage overhead.

### 1.2. Issue and Overview of Our Solution

Crowdsensing applications generally need a large number of mobile devices to sense the required data. It is difficult to obtain a sufficient amount of data by solely relying on voluntary participation. Because it uses workers’ computational resources and time to sense the required data. Therefore, rewarding workers is a positive incentive mechanism to attract more participants. On the one hand, requesters need sensing data that meet the requirements. On the other hand, workers need fair payments that correspond to data quality. If the crowdsensing platform cannot guarantee fairness, workers lose their incentive to participate. Therefore, the guarantee of fair dealing for a crowdsensing application is essential.

However, the requirements of sensing data are created by requesters. Malicious requesters can violate fairness in the following way. The requesters purposefully create an abnormal requirement to cause sensing data to fail the quality evaluation, even though the actual quality is sufficiently high. Then, they can control a miner or a full node to gain access to the sensing data without paying a reward to workers. The motivation of malicious requesters is clearly obvious because requesters obtain the sensing data without the need to pay. Furthermore, because the blockchain assists requesters in becoming anonymous, malicious requesters can simply change their blockchain addresses; this enables them to continue implementing these malicious actions by effectively remaining anonymous and without consequence. This is clearly an important vulnerability to address to support the motivation for workers to participate. Workers are also concerned about privacy leakage once sensing data are uploaded to the blockchain, and requesters want to avoid releasing sensing data to the public if the data contain sensitive information, such as trade secrets. It is a requirement that only the requester and the worker have access to the data. However, current proposed blockchain-based crowdsensing systems that execute smart contracts to evaluate sensing data do not meet the requirements for data confidentiality.

To address the issues mentioned above, we leverage Trusted Execution Environment (TEE) on blockchain-based crowdsensing and propose a novel scheme. However, a simple use of TEE is not sufficient to address the issues because the secure area of TEE, called the enclave, is designed to guarantee code and data loaded inside to be protected with respect to confidentiality and integrity. It cannot prevent requesters from submitting a malicious smart contract into an enclave. The key idea of our proposal in this paper is that if and only if the sensing data uploaded by workers meets the requirements and the workers get paid, the sensing data can be stored outside the safety boundary of the enclave. To achieve this, we execute a smart contract of the requirements inside an enclave, along with the sensing data uploaded by workers through a Transport Layer Security (TLS) [14] connection. If the sensing data meet the requirements, another smart contract will be executed to pay the worker by using the requester’s deposit. After the smart contract inside the enclave verifies that the worker gets paid successfully, the sensing data can be stored in the blockchain, which is outside of the safety boundary of the enclave. If the sensing data do not meet the requirements or the enclave fails to verify the payment, this crowdsensing transaction will be terminated. For data confidentiality and integrity, the enclave encrypts the data and calculates a digest of the ciphertext, then signs the digest before storing it outside the safety boundary of the enclave.

The contributions of this paper are as follows:

- We propose a novel scheme for blockchain-based crowdsensing applications to solve the problem of malicious requesters tricking workers by publishing abnormal sensing data requirements. The scheme guarantees the fairness of crowdsensing transactions. Requesters obtain the sensing data that meet the requirements but receive nothing if they do not pay workers, while workers need not to verify the requirement contract without concerning about being tricked.
- We present a privacy-preserving solution that ensures the sensing data can only be accessed by the worker and corresponding requesters, regardless of the data being processed through a smart contract executing in the blockchain.
- We investigate secure and efficient solutions to determine whether an enclave validates payments in the case of our proposed scheme.

The remainder of this paper is structured as follows. In Section 2, we present related works of blockchain-based crowdsensing. In Section 3, we present the brief introduction of blockchain, smart contract and Trusted Execution Environment. In Section 4, we first formulate the problem addressed in this study. Then, we present our proposed scheme to improve fair dealing in crowdsensing for both requesters and workers, and we explicate a solution to secure sensing data throughout crowdsensing transactions. In Section 5, we describe our experimental setup and results. In Section 6, we discuss the fairness, security, on-chain and off-chain TEE of our scheme. Finally, we conclude the paper with a brief summary of our contributions in Section 7.

## 2. Related Work

Recently, researchers have focused on applying blockchain to crowdsensing applications in an effort to address these challenges. However, to date, only a few contributions have been made. Wang et al. [15] proposed a blockchain-based privacy-preserving incentive mechanism in crowdsensing applications. In this system, a blockchain network plays the role of a trusted third party. It is responsible for evaluating the quality of sensing data and the payment for workers providing the data. The paper also proposes intra-group negotiation and k-anonymity privacy protections. Jia et al. [16] presents a crowdsensing system focusing on solving the issue of sensing data leaking workers’ location privacy when they upload data to the blockchain. Li et al. [17] presents a blockchain-based decentralized framework for crowdsourcing mainly to solve the problem of a single point of failure in the traditional crowdsourcing system. These valuable contributions solve problems regarding reliability, privacy-preservation, and security. However, the challenge of requesters publishing malicious requirements to the blockchain to trick workers in existing crowdsensing systems has not been discussed. To the best of our knowledge, this is the first paper to focus on addressing this pertinent issue in blockchain-based crowdsensing applications.

Bowman et al. [18] proposed private data objects (PDO), which enables a privacy-preserving approach to run smart contracts over private data by leveraging Intel Secure Guard Extensions (SGX) [19,20]. However, smart contracts are provided by a trusted party, and the user must communicate with the contract maker off-chain to verify and approve the contract before using it. In contrast, our proposal avoids verifying the requirement contract created by requesters but can still guarantee the fairness of crowdsensing transactions. Fairswap [21] is an efficient protocol for fair exchange of digital goods using smart contracts and blockchain. However, the protocol bases on the fact that the buyer has already known the hash digest or identity of the digital goods, in order to verify the goods sent by the seller is the exact one. This is not suitable for our case, because a requester do not know what data is owned by the worker in advance.

## 3. Preliminaries

### 3.1. Blockchain and Smart Contract

A blockchain is an open, distributed ledger that can record transactions among different parties in a verifiable and permanent way [22]. The whole point of using a blockchain is to let people — in particular, people who do not trust one another — share valuable data in a secure, tamperproof way [23]. A smart contract [11] is a computer protocol intended to digitally facilitate, verify, or enforce the negotiation or performance of a contract. Smart contracts allow the performance of credible transactions without third parties. These transactions are trackable and irreversible. In blockchain, we can also call a smart contract as Chaincode, which is the term for programs that run on top of the blockchain to implement the business logic of how applications interact with the ledger. A new publishing transaction can trigger chaincode that decides what state change should be applied to the ledger.

### 3.2. Trusted Execution Environment (TEE) [24]

The definition of TEE as presented in Reference [24] is as follows:

TEE is a tamper-resistant processing environment that runs on a separation kernel. It guarantees the authenticity of the executed code, the integrity of the runtime states (e.g., CPU registers, memory, and sensitive I/O), and the confidentiality of its code, data, and runtime states stored on a persistent memory. In addition, it shall be able to provide remote attestation that proves its trustworthiness for third-parties. The content of TEE is not static; it can be securely updated. The TEE resists against all software attacks as well as the physical attacks performed on the main memory of the system. Attacks performed by exploiting backdoor security flaws are not possible.

Enclave protection. The secure area of TEE is called an enclave, which is a CPU-protected address space that is accessible only by the code within the enclave. A machine consists of two parts, untrusted environments and secure enclaves. The initial code and data in an enclave are loaded by untrusted system software in the untrusted environment. Each enclave is identified by a measurement of its code and metadata at build time. Thus, two enclaves with the same measurement are identical. CPU performs the measurement and signs the measurement with its private key. A duplicate measurement is made at enclave load time and the results are compared with the original signed measurement to detect whether any changes were made to the released enclave. The signature of the CPU is used to check the integrity of the measurement.

Remote attestation. A Trusted Platform Module (TPM) is a tamper resistant piece of cryptographic hardware built onto the system board that implements primitive cryptographic functions and signs the measurement hash with its private key. A remote party can undergo a software attestation process and verify the signature with the help of an Attestation Service, such as Intel Attestation Service (IAS), to convince itself that it is communicating with an enclave that has a specific measurement hash, and is running in a secure environment.

Our implementation uses Intel Software Guard Extensions (SGX) to provision the TEE. However, it is noteworthy that our design works with other TEE instantiations (e.g., TrustZone [25], Sanctum [26]).

## 4. Blockchain-Based System Model

### 4.1. Problem Formulation

**Figure 1:**
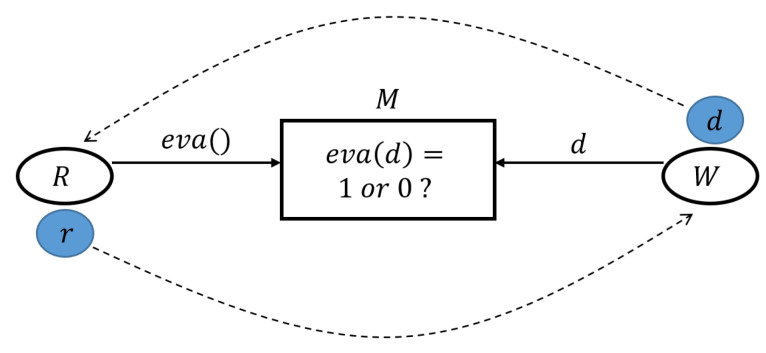
Relationships among Requesters *R*, Workers *W* and blockchain miners *M*.

People who require specific sensing data, called requesters *R*, can publish their requirements *eva*() and declarations of rewards to a crowdsensing application platform. People in possession of the sensing data *d* that meet the requirements, called workers *W*, can provide this data to requesters and receive a reward *r* (Figure 1). However, these two parties do not trust each other. *W* is concerned that after uploading *d*, *R* refuses to send them *r*, while *R* is concerned that *d* does not meet their requirement after paying *W* using *r*.

Therefore, to eliminate concerns, a blockchain network, comprising many miner nodes *M*, is responsible for evaluating *d*, denoted by *eva*(*d*), and to pay the workers automatically and mandatorily if *d* meets the requirement, that is, *eva*(*d*) = 1. However, because of the openness and transparency of *d* stored in the blockchain, everyone can access *d* as soon as *W* uploads *d* to the blockchain. Here, the problem is that *R* can also act as a *M* or can control a *M* to access *d* before executing *eva*(*d*). In this case, a malicious *R* can create and publish an abnormal *eva*() that always ensures *eva*(*d*) = 0. This represents an example of unfair dealing because *R* receives *d* without losing *r*, but *W* loses *d* without receiving *r*.

### 4.2. Overview Model of FairCs

**Figure 2:**
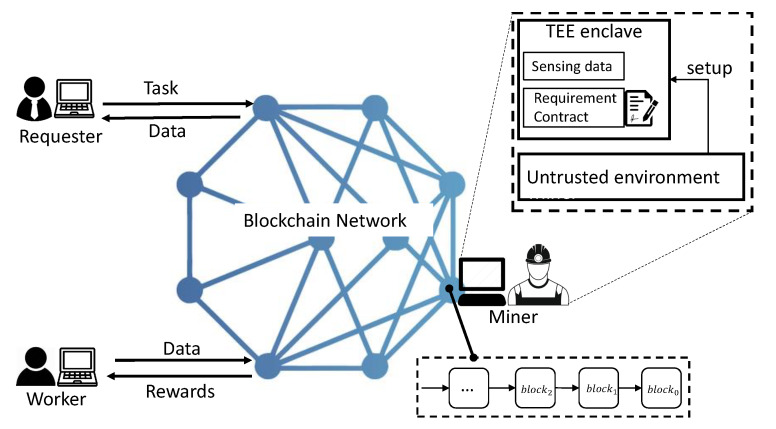
Overview of the crowdsensing system based on blockchain and Trusted Execution Environment (TEE).

Four main parties are involved in the system shown in Figure 2, these are miners, requesters, workers and a blockchain network. Miners are in charge of collecting, validating transactions and creating blocks for the blockchain. They gain mining rewards and transaction fees if they succeed in creating a block. A miner can become a requester to publish tasks or a worker to complete tasks. A miner node consists of two parts mainly, an untrusted environment and secure TEE enclaves. Requesters, who need sensing data, publish a task to the blockchain. They pay workers who can upload sensing data to the blockchain if the data meets their requirements. A task consists of a reward deposit and a sensing data requirement that is codified as a smart contract (i.e., chain code). Workers are persons who accept the task published by requesters in the blockchain. They collect sensing data by using specified mobile devices and upload the data to the blockchain, and then receive rewards. In our system, the blockchain network, consisting of a great many miner nodes, provides a crowdsensing service, responsible for executing evaluation *eva*(*d*), executing reward payment, and storing sensing data.

### 4.3. Blockchain-Based Fair Crowdsensing Scheme (FairCs) Using TEE

**Figure 3:**
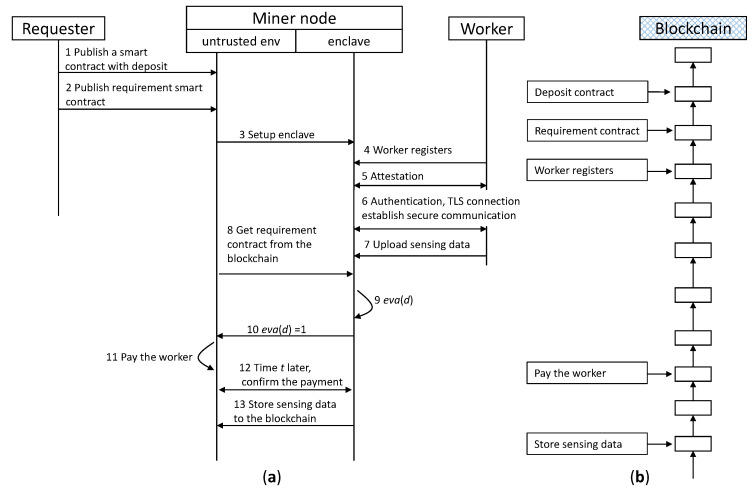
(**a**) Operation sequence of our proposed crowdsensing scheme, (**b**) Transactions that are added to the blockchain at the time corresponding to the operations of the left sequence.

Figure 3 shows the operations of our proposed crowdsensing scheme. (Steps 1–2) The requester submits a smart contract with a deposit before publishing a requirement contract *eva*() to the blockchain. The deposit represents the reward *r* to guarantee that the requester is able to pay workers if *eva*(*d*) = 1. (Steps 3–7) Then, a secure enclave is set up by the untrusted environment in the miner node. The worker performs remote attestation [27] with the enclave residing on the miner node to gain confidence that the intended software is securely running within an enclave on a TEE enabled platform. If the attestation passes, the worker establishes secure communication with the enclave through the TLS connection and uploads sensing data to the enclave. The enclave concurrently gets the requirement contract published by the requester from the blockchain. (Steps 8–11) The miner node executes the requirement contract with the sensing data (i.e., *eva*(*d*)) inside the enclave to evaluate the data. If the data meet the requirements, the enclave outputs 1 (i.e., *eva*(*d*) = 1) to the untrusted environment of the miner node, which is responsible for paying rewards to the worker using the requester’s deposit. Otherwise, if *eva*(*d*) = 0, the enclave deletes the sensing data and terminates the transaction. (Steps 12–13) After a period of time t, the enclave validates the payment and stores the sensing data in the blockchain. The value of time t is a parameter that depends on the blockchain security strategy, for example, 60 min in Bitcoin. Finally, if payment validation fails, the enclave will delete the data and terminate the transaction.

Our proposed scheme provides a simple way to secure privacy over the sensing data. The data are not secure if they are stored outside the safety boundary of the enclaves. All miner nodes have the sensing data inside the enclaves, and to achieve data confidentiality and integrity, the enclave secure the data before storing them outside the enclave. Specifically, the enclave first encrypts the sensing using the public key of the requester, then computes a hash value of the ciphertext and sign the value using the enclave’s private key. Finally, attach the signature to the ciphertext. The requester can download the data from the blockchain and decrypt the data using the private key. Then, the hash values of the sensing data are calculated, which are then compared to validate the integrity of the data.

We present the pseudo-code of the smart contract for requirements as follows (Algorithm 1). Function *EVALUATE_SENSING_DATA*(*sensingData*) is a function interface that all requesters must implement. Requesters must finish the logic code to evaluate the sensing data based on their own needs. If the sensing data meet the requirement, return 1, else return 0.
**Algorithm 1:** Smart contract of requirement published by requesters**Input:**sensingData**Output:** 1, if the sensing data meet the requirement; else return 0. 1: **function**
evaluate_sensing_data(sensingData) 2:    Logic code of evaluating sensingData 3:    **if**
sensingData meets the requirement **then** 4:        **return** 1 5:    **else** 6:        **return** 0 7:    **end if** 8: **end function**

### 4.4. Payment Validation by Enclave

**Figure 4:**
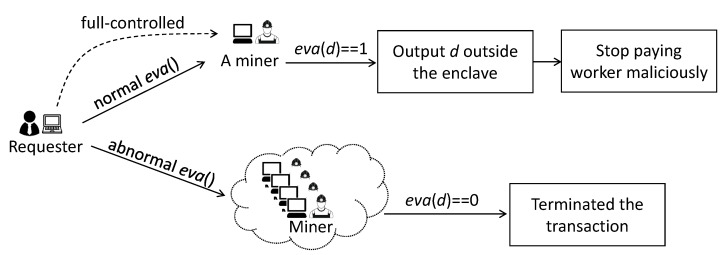
Requester performs a trick if the enclave does not validate payment before storing the sensing data outside the enclave.

The payment validation (i.e., step 12 in Figure 3), which occurs before storing sensing data outside the safety boundary of the enclave, is very necessary. Otherwise, malicious requesters can perform the following type of trick (Figure 4). First, the requester publishes an abnormal requirement contract (i.e., *eva*()) to the blockchain network. This requirement contract always returns *eva*(*d*) = 0, causing the nodes in the blockchain network to not pay the worker. However, then, the requester creates a new but normal *eva*() and fully controls one miner node to execute this *eva*(). If *eva*(*d*) = 1, the sensing data *d* will be stored outside the enclave as expected. Since the requester can fully control the miner node, it can access the sensing data as soon as they are outputted from the enclave, then it stops paying the worker once the data are accessed. In this case, the worker loses their sensing data without being rewarded. Therefore, it is necessary to verify the reward payment before storing the data outside the safety boundary of the enclave.

**Figure 5:**
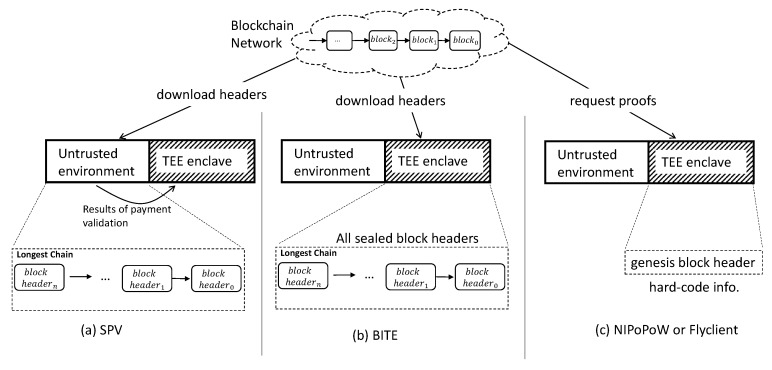
Payment validation approaches performed by enclaves. (**a**) Simplified Payment Verification (SPV) downloads all block headers from the blockchain network and maintains them in the untrusted environment. The TEE enclave gets results of payment validation from the untrusted environment. (**b**) Bitcoin Lightweight Client Privacy using Trusted Execution (BITE) also downloads all block headers from the blockchain network. However, it maintains them inside the safety boundary of the TEE enclave via a sealing technique. Thus, the enclave can validate payments itself. (**c**) Non-Interactive Proofs of Proof-of-Work (NIPoPoW) or Flyclient only requests proofs from the blockchain network. They do not need to download and maintain all block headers locally. The genesis block header (i.e., the first header of a blockchain) is hard coded in the enclave to help verify the proofs.

There are several approaches that an enclave can use to validate payments, as shown in Figure 5; these are Simplified Payment Verification (SPV) [28], Bitcoin Lightweight Client Privacy using Trusted Execution (BITE) [29], Non-Interactive Proofs of Proof-of-Work (NIPoPoW) [30], and Flyclient [31]. SPV validates a transaction by downloading all block headers to the local storage in the untrusted environment of a miner node. It is unsecure for an enclave to validate payments because the node owner, who could be a requester, can fully control the node and tamper with the storage arbitrarily and maliciously. To prevent the node owner accessing and tampering with the block headers, BITE proposed a method that maintains all block headers inside the safety boundary of the enclaves by using a sealing [27] technique provided by a TEE. Therefore, it is a more suitable and secure solution to validate payments in our case. However, some drawbacks include the creation of a high payload for the CPU to maintain the block header structure, as well as a large amount of local storage to store all block headers. NIPoPoW provides a non-interactive way for a light client to validate a transaction. The client does not need to download and maintain all block headers locally. However, this works only for Proof of Work (PoW) consensus [28], and also only if a fixed PoW difficulty is assumed for all blocks. Flyclient is a novel solution that addresses some drawbacks of NIPoPoW and improves the efficiency of chain commitments, and non-interactive and transferable proofs. However, one of the drawbacks of NIPoPoW or Flyclient is that it requires special servers that equip the corresponding service of NIPoPoW or Flyclient in order to respond to requests of proofs for payment validation. In conclusion, BITE and Flyclient are the most secure and suitable solutions for enclaves to validate payments in our proposed scheme.

### 4.5. Threat Models

The attacker or malicious requester has full control of a miner node; it can read and write to the memory of any running process, even the operating system. It can modify data on disk, intercept and change the content of any system call. It can modify, reorder and delay network messages arbitrarily. It can start, stop and invoke the local TEE enclaves with an arbitrary input. We assume that the attacker cannot break the hardware security enforcements of TEE. That is, the attacker cannot compromise the integrity and confidentiality of protected enclaves. It can execute arbitrary smart contracts in a small amount of nodes, but cannot corrupt the majority of nodes on the blockchain network to perform a 51% attack.

## 5. Experiment and Results

### 5.1. Experimental Setup

We leverage Intel SGX as a TEE included in recent Intel CPUs which introduces the concept of isolated hardware enclaves that can be created and managed using new CPU instructions. The experimental setup consists of three machines, which are all connected through the Internet, but in one room using the same switch. First, we use an Intel SGX-enabled machine running Ubuntu 18.04 LTS with an Intel Xeon processor E3 CPU clocked at 3.80 GHz and 24 GB RAM, where we install an Ethereum [32] client Ganache [33] to build a personal Ethereum blockchain locally with some modifications to fit our case of FairCs, OpenEnclave [34] as a Software Development Kit (SDK) for building the enclave under Intel SGX hardware, Microsoft eEVM [35] as a standalone Enclave ready Ethereum Virtual Machine running inside an enclave to execute smart contracts. This machine acts as a miner node. In addition, we implement a server in C++ to receive and respond to requests from workers and requesters. This server runs in this node and interacts with the Ethereum client via a JSON RPC API [36]. TEE enclaves run on this server as security areas. Second, a machine running Windows 10 Pro on an Intel Core i5-8500 CPU clocked at 3.00 GHz with 32 GB RAM, plays the role of the worker. Third, a machine running Windows 10 Home on an Intel Core i7-4790 CPU clocked at 3.60 GHz with 32.0 GB RAM, plays the role of the requester.

### 5.2. Naive Version of Blockchain-Based Crowdsensing Application

**Figure 6:**
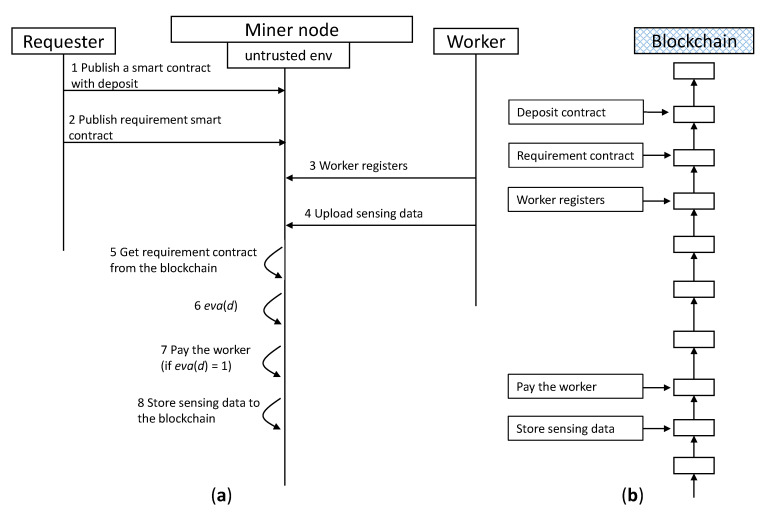
The naive version of blockchain-based crowdsensing operations without using TEE, comparing with the one in Figure 3. (**a**) Operation sequence of an insecure crowdsensing scheme, (**b**) Transactions that are added to the blockchain at the time corresponding to the operations of the left sequence.

Figure 6 shows a naive version (NV) of blockchain-based crowdsensing operations without using TEE to guarantee security and fairness. If all parties are trustworthy, this NV can achieve the same functionalities as FairCs. That is, the difference between the NV and FairCs is that the NV does not use TEE, attestation and TLS connection. Thus, as soon as the sensing data are uploaded to the miner node, the data becomes public and transparent, which can be accessed by everyone. In addition, the NV does not need to wait for time *t* for payment validation if *eva*(*d*) = 1, and the sensing data are stored in the blockchain immediately.

We present the NV here because we can evaluate how much additional overhead that the TEE enclave brings in, by comparing the performance between the FairCs and the NV. Comparing the operations between Figure 3 and Figure 6, the transactions related to the requester and the worker are exactly same, as well as the transactions that are added to the blockchain. That is, FairCs does not affect the operation of the requester nor does it influence the worker to participate in a crowdsensing task, even though it introduces TEE for security and fairness.

### 5.3. Results

**Figure 7:**
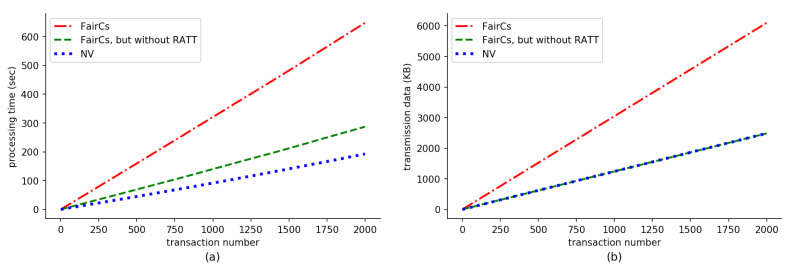
Evaluation results of (**a**) processing time and (**b**) transaction data with a varying number of transactions.

We evaluate processing time and network transmission data of the FairCs compared with the NV shown in Figure 7. The x-axis of the figure represents the transaction number. Note that (1) a transaction here is from the time that a requester publishes a deposit smart contract to the time that the sensing data are stored in the blockchain, that is, it includes all steps in Figure 3; (2) to exclude the impact of the sensing data size, we use a very small and negligible size of sensing data for this experiment; (3) on the left of Figure 7, we only consider the processing time of the server in a miner node, rather than the processing time of the clients of requesters or workers. Furthermore, because our experiment uses a personal blockchain network, it is no need to wait for about 15 s of mining time. So a valid transaction can be accepted and mined into a block immediately. FairCs uses TEE, remote attestation and TLS. This explains why the processing time of FairCs is longer than the case of the NV for each transaction. If we remove Remote ATtestation and TLS (RATT) from our proposal, the processing time is illustrated by the middle line in the left sub-figure, which is very close to the line at the bottom. Thus, we can establish a conclusion that the processing involved for RATT requires much more CPU resources and creates the most additional CPU overhead for FairCs when compared with the NV. In the sub-figure (**b**) of Figure 7, we present the transmission data of the FairCs, shown by the top line. If we remove RATT from the system, the transmission data are the same as the NV, as was expected. This is because the operation between workers and miners or between requesters and miners is the same for both the FairCs and the NV. Thus, from this sub-figure, we can conclude that RATT generates all network traffic overhead for our proposed system.

**Figure 8:**
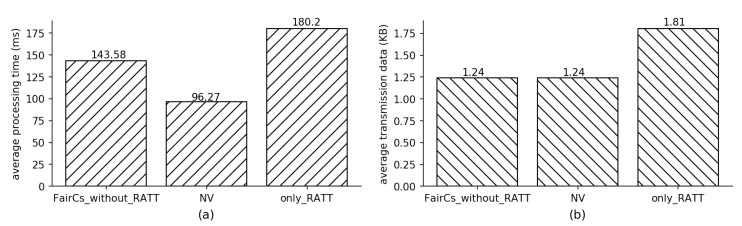
Experiment results of (**a**) average processing time of (left) FairCs but without equipping Remote ATtestation and TLS (RATT), (middle) naive version (NV) and (right) only RATT for each transaction. (**b**) average size of transmission data for each transaction.

Furthermore, we calculate the average of processing time and communication transmission data for each transaction to demonstrate the performance influence of using TEE. Figure 8 shows the results. Our FairCs equips the components of TEE and RATT to guarantee security. In Figure 8, the label “FairCs_without_RATT” means the case that FairCs does not equip the component of RATT; the label “only_RATT” means the time or transmission data size is brought in by only RATT. We calculate their total processing time and transmission data size over 2000 transactions by using the way mentioned in the previous paragraph, and further divide them by 2000 to get their average values. The results show that the processing time and network transmission data of the part where we only use RATT is greater than the other two, which summarizes that RATT creates the most performance overhead of FairCs when compared with NV.

**Figure 9:**
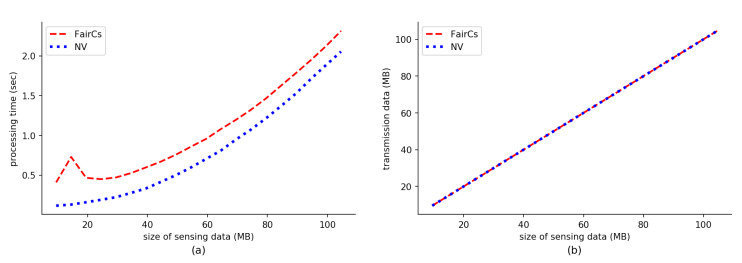
Evaluation results of the system performance with different sizes of sensing data. (**a**) Processing time of FairCs and NV. (**b**) Network transmission data size.

Figure 9a illustrates the impact of different sizes of sensing data on the FairCs and the NV. The larger the sensing data, the longer the processing time of the server. Note that the waiting time for transactions to be accepted by the blockchain is not included. Figure 9b illustrates that the network transmission data size between the worker and the miner or the requester and the miner. The data size is almost the same between both the FairCs and the NV. The reason for this is that the FairCs and the NV both have the same transaction procedures, except for the operation of RATT for the FairCs. In addition, the network transmission payload of a RATT operation is about 2 KB, which is much smaller than the size of the sensing data.

## 6. Discussion

**Fairness.** FairCs overcomes the issue that malicious requesters can obtain sensing data without paying workers by creating abnormal sensing data requirements. First, if the requester is not a miner of the blockchain, they can only access the sensing data after the data are stored in the blockchain. In this case, the requester can obtain the data that meet his/her requirements but will receive nothing if the data do not. The worker does not need to concern themselves about being tricked by the requester who distributes a malicious requirement contract. Second, if the requester is also a miner of the blockchain, TEE guarantees that the miner can only access the sensing data after the execution of *eva*(*d*) and only if *eva*(*d*) = 1 because (1) the attestation of TEE validates that the remote enclave is set up properly and (2) the worker communicates with enclave through a TLS connection to guarantee data security during the network transmission. Regardless of the fact that the requester (currently also a miner) can fully control their miner node to not pay the reward even if *eva*(*d*) = 1, the enclave in this node will never output the sensing data or store them in the blockchain, because of the failure of the payment validation. FairCs allows workers to avoid verifying requester’s requirements, which means it is still considered fair for workers even though the requirements are malicious. Workers do not need to worry about being tricked by requesters who create malicious requirements. This guarantees that the requester can obtain sensing data only if it pays the workers.

**Sensing data security.** The sensing data can only be accessed by the requester and the worker, even though the data are evaluated by a smart contract in the blockchain network. First, the workers perform remote attestation to gain confidence that the intended software is securely running within an enclave. Then, a TLS connection is established before uploading the sensing data to the enclave. The data security inside an enclave is guaranteed by the TEE. Thus, the data are secure when the requirement contract is executed inside the enclave for evaluation. Second, the sensing data are encrypted using the public key of the requester before being stored outside the secure boundary of the enclave, and can only be decrypted by the corresponding requester using its private key. The requester can validate the data integrity through the signature signed by the enclave. In short, the key point is that the sensing data in our system are secure and can only be accessed by the requester and the worker.

Requesters can access the sensing data uploaded by workers. Thus, if the sensing data contain private information such as the worker’s location, to protect this privacy against the requester, we must use other techniques; this is out of the scope of this paper.

**On-chain and off-chain.** Practically, the fact that every node in the blockchain network is equipped with TEE is not realistic. On the one hand, TEE needs specified hardware support, such as Intel SGX and ARM Trustzone, which produces an unexpected additional expense for miners. On the other hand, the performance of software running in a TEE is slower than non-TEE environments. In addition, validating sensing data through peer-to-peer networking and storing sensing data in the blockchain will create significant overhead for the whole blockchain network with respect to network traffic and storage. Especially, in general practice, the size of sensing data is large. One way to solve the issue is to apply off-chain TEE, which does not require that all miner nodes are equipped with TEEs, and the sensing data need not be broadcasted through the blockchain network. In addition, the data do not need to be stored in the blockchain. This requires that only a few TEE-equipped nodes are responsible for validating the sensing data and performing the payments of rewards. Those nodes generate proofs of the evaluation results over sensing data, the digests of the sensing data and so forth. Then, these status-based data are published and stored on the blockchain network. The miner nodes on the blockchain network verify the proofs and create blocks including those valid status-based data.

## 7. Conclusions

Motivation to participate in crowdsensing tasks can be significantly encouraged if the crowdsensing service platform can provide high anonymity, privacy preservation and fair dealing. This study focuses on solving the issue with respect to fair dealing by leveraging TEE and blockchain techniques. We propose a novel crowdsensing scheme to overcome the issue that malicious requesters publish abnormal sensing data requirements to trick workers into not being paid for providing sensing data. Our experiment shows that our scheme does not generate much overhead but can improve fair dealing in crowdsensing applications.
